# Optimization of Enzymatic Transesterification of Acid Oil for Biodiesel Production Using a Low-Cost Lipase: The Effect of Transesterification Conditions and the Synergy of Lipases with Different Regioselectivity

**DOI:** 10.1007/s12010-024-04941-3

**Published:** 2024-05-02

**Authors:** Alexandra Moschona, Androniki Spanou, Ioannis V. Pavlidis, Anastasios J. Karabelas, Sotiris I. Patsios

**Affiliations:** 1 Laboratory of Natural Resources and Renewable Energies, Chemical Process and Energy Resources Institute, Centre for Research and Technology – Hellas, Thermi, Thessaloniki, Greece; 2 Department of Chemistry, University of Crete, Heraklion, Greece

**Keywords:** Transesterification, Second-generation biodiesel, Lipases, Biolipasa-R, Immobilization, Acid oil

## Abstract

This study describes the enzymatic production of second-generation biodiesel using low-quality acid oil as a substrate. Biolipasa-R, a commercially available and low-cost lipase, was employed for enzymatic transesterification. Response surface methodology was applied to optimize the enzymatic transesterification process. The optimal conditions for biodiesel production, which comprised 42% lipase concentration (per weight of oil), 32% water content (per weight of oil), a methanol to oil molar ratio of 3:1, pH 7.0 and reaction temperature 30°C, resulted in the highest fatty acid methyl ester (FAME) content (71.3%). Subsequently, the synergistic effect of two lipases with different regioselectivities under the optimum transesterification conditions was studied, aiming at the enhancement of process efficiency. The transesterification efficiency of immobilized Biolipasa-R was determined and compared to that of Biolipasa-R in its free form. The results revealed a good performance on FAME content (66.5%), while the recycling of immobilized lipase resulted in a decrease in transesterification efficiency after three consecutive uses.

**Supplementary Information:**

The online version contains supplementary material available at 10.1007/s12010-024-04941-3.

## Introduction

Biodiesel is a mixture of long-chain fatty acid methyl esters (FAME) that are rather biodegradable and non-toxic, and their properties are similar to those of light petroleum oil; therefore, it is suitable for use in diesel engines [1]. It is mainly produced from vegetable oils or animal fat through the process of transesterification [2]. This process involves converting triglycerides into monoesters and is conducted in the presence of various types of catalysts (i.e., basic, acidic, and enzymatic), depending on the process employed [3]. In response to the necessity of mitigating the environmental impact associated with oilseeds usage, such as deforestation of tropical forests, loss of biodiversity, and the reduction of global food reserves, the European Parliament has recently voted in favor of revising the Renewable Energy Directive (REDII). This revision encompasses two primary objectives: first, the gradual elimination of 1^st^ generation biofuels by the year 2030, and second, the establishment of a target for 14% of transport fuels to be sourced from second-generation biofuels [4]. These second-generation biofuels are derived from raw materials that are unsuitable for use as food, including agricultural, forest, industrial, and urban residues. Through the implementation of these measures, the revised EU Directive aims to promote more sustainable and eco-friendly fuels for the transport sector while curbing the adverse impacts linked to conventional oilseed-based biofuels [5].

The cost of the raw material is a crucial aspect of biodiesel production, corresponding to at least 80% of the total cost of biofuel production [6]. Therefore, various studies focus on the utilization of less expensive feedstocks, like non-edible oils, waste cooking oils, and animal fats [7–10]. However, these feedstocks have certain drawbacks, primarily attributed to their high content of free fatty acids (FFA), which are not suitable for the commonly employed homogeneous base-catalyzed transesterification process due to the formation of soap through the reaction of FFA with the catalyst, leading to catalyst deactivation [11]. Considering also the drawbacks associated with conventional transesterification processes (i.e., challenges with glycerol recovery and purification, the difficulty of removing basic catalysts and water from biodiesel, the generation of a significant amount of wastewater, and other issues such as emulsification) biodiesel production through enzymatic transesterification appears highly promising [12, 13].

Enzymatic catalysis has received increased attention for biodiesel production, mainly using commercially available enzyme products and oils of different origins [14–17]. Compared with the commonly applied base catalysts, enzymes can catalyze the reaction in the presence of water and high FFA content [18, 19]. Many studies have contributed to and analyzed in depth the transesterification mechanisms and kinetics of specific enzymes, as well as novel classes of enzymes and oils for the biocatalytic synthesis of first- and second-generation biodiesel [20–22]. Enzymatic transesterification offers several other major advantages, including relatively simple operations, low methanol consumption, easy recovery of produced glycerol, mild reaction conditions, and the absence of chemical catalyst and wastewater production, which contribute to process sustainability and cost-effectiveness [11, 23, 24].

Enzymes/biocatalysts (lipases) are primarily derived from bacteria (i.e., *Burkholderia cepacia, Pseudomonas mendocina*) or fungi (i.e., *Candida antarctica, Rhizopus oryzae*) and are the most widely used catalysts, not only in the chemical, food, and pharmaceutical industry but also in biodiesel production [25, 26]. The use of lipases allows transesterification of triglycerides present in the raw material forming alkyl esters. In terms of regioselectivity, lipases could be divided into three categories: (a) sn-1,3-selective (hydrolyzing the ester bonds of triglycerides at positions C1 or C3), (b) sn-2-selective (hydrolyzing the ester bonds of triglycerides at position C2) and (c) non-selective (acting on random positions in the ester bonds of triglycerides converting them into fatty acids and glycerol) [19, 27]. A synergistic effect occurs as a result of the distinct regioselectivities of lipases. Thus, when combining a 1,3-regioselective lipase with a non-regioselective lipase, there is a possibility of enhancing the performance of both lipases through this synergistic effect [12, 13, 28, 29].

Nevertheless, there is an important disadvantage concerning enzymatic transesterification related to its high cost attributed to the use of enzymes. While the cost of sodium methoxide, used as a catalyst in basic transesterification is approx. 4–5 €/kg of active substance, the cost of commercial lipases may vary between 100 and 10,000 €/kg. The quantity of the enzymes required and their high price highlight the need for the development of suitable technologies to recover and reuse these enzymes. Nonetheless, the recovery and separation procedure from the reaction mixture may be intricate [30, 31]. Moreover, the reusability of the recovered enzymes is limited since their efficiency gradually diminishes after each reuse cycle. Immobilized lipases could be used to partially overcome these drawbacks. They can be immobilized within suitable natural or synthetic carriers, offering the advantage of easy separation from the reaction mixture, and can be used in multiple reaction cycles with less activity loss. Beyond the benefits of recovery and reuse, immobilization plays a crucial role in stabilizing lipases under conditions (i.e., organic solvent, high or low pH, and temperature) that would be challenging to the lipase in its free form [32].

The examination of the scientific literature underscores the increasing interest in enzymatic transesterification as a sustainable and environmentally friendly alternative to conventional chemical processes [6, 14, 23]. Notably, researchers have explored the utilization of inexpensive raw materials, particularly in light of the global food crisis, with used cooking or frying oil emerging as an attractive feedstock for biodiesel production, simultaneously addressing waste management concerns [33, 34]. Binhayeeding et al. [12] investigated a mixture of immobilized lipases of different origins (i.e., *C. rugosa* and *R. miehei*), for the transesterification of waste cooking oil to produce FAMEs. Under optimal conditions (enzyme loading 1%, 1:1 ratio of lipases, 5% water, a methanol-to-oil ratio of 6:1, and reaction temperature of 45°C), they reported a FAME yield of 96.5% after 24 h. Moreover, Li et al. [35] achieved a biodiesel yield of up to 94% using recombinant *Rhizopus oryzae* lipases immobilized on macroporous and anion exchange resins for the transesterification of renewable non-edible *Pistacia chinensis Bunge* seed oil with methanol, observing no obvious loss in biodiesel yield after the immobilized lipase was consecutively used for five cycles, under optimum conditions (methanol to oil molar ratio 5:1, water content 20% by weight of oil and temperature 37°C). In another research, Poppe et al. [36] evaluated the synergistic effect of lipases for biodiesel production using soybean oil and waste frying oil. Using soybean oil, the optimum reaction conditions were found to be a molar ratio of ethanol:soybean oil of 4.95:1 and combi-lipase composition of 10% *Thermomyces lanuginosus* lipase, 75% lipase B from *Candida antarctica*, and 15% *Rhizomucor miehei* lipase, while using waste oil, the optimum conditions were a molar ratio of ethanol:waste oil of 9:1, and a combi-lipase composition of 20% *Thermomyces lanuginosus* lipase, 60% lipase B from *Candida antarctica*, and 20% *Rhizomucor miehei* lipase [36]. The innovation of this research was that transesterification was conducted under ultrasound, with the highest biodiesel yields achieved being 95% using soybean oil and 71% using the waste frying oil [36]. Yasvanthrajan et al. [37] also evaluated the transesterification of waste cottonseed oil catalyzed by immobilized *Rhizopus oryzae* lipase, noting a 50% improvement in conversion with the presence of ultra-sonication compared to conventional processes. Research efforts have been made to enzymatically catalyze the transesterification of non-edible, such as jatropha oil [38, 39], waste rapeseed oil [16], acidic oils of macauba [40] and canola waste cooking oil [25], achieving FAME yields of up to 80%. Efforts to overcome the major drawback of enzymatic transesterification, which is the high enzyme costs, have led to investigations into novel enzymes, such as cutinases [41] and acyltransferases [42], as well as whole-cell biocatalysts [19, 43], offering promising results for improving biodiesel production efficiency. For instance, Yang et al. [44] demonstrated improved biodiesel production from tallow seed oil by co-displaying *Candida rugosa* lipase and *Rhizopus oryzae* lipase on the whole-cell catalyst of *Pichia pastoris*. In another work, Balasubramaniam et al. [45] compared biodiesel production using waste cooking oil as substrate, catalyzed by a lipase produced by the fungi *Rhizopus oryzae* 262, and a commercially available pure lipase enzyme, both immobilized on calcium alginate beads. They found that the FAME yields of the whole-cell and the pure lipase enzymes were 84 and 94%, respectively, at 30 °C after 24 h (methanol to oil molar ratio 3:1 and amount of enzyme 10% w/w_oil_) [45].

In this context, the present work focuses on the enzymatic transesterification of low-quality acid oil for the production of second-generation biodiesel using a low-cost, commercially available lipase, aiming at the economic viability of the enzymatic process. Acid oils are non-edible oil generated by the chemical refining of edible oils in biodiesel production industries. Their utilization aligns with the Renewable Energy Directive (REDII), reduces the overall cost of biodiesel production, and eliminates expenses associated with waste disposal. They are deemed suitable according to criteria related to global warming impact and energy demand [46]. We employ Biolipasa-R, a rather inexpensive lipase produced by Biocon (Biocon S.L., C/Bélgica, Spain), which is utilized in the food industry. It is a powder-formulated enzyme that contains *Rhizopus oryzae* lipase (ROL), and its commercial price (retail sale in 2022) is approx. 0.10 €/g of lyophilized powder; the main specifications of Biolipasa-R are summarized in Table 1. Biolipasa-R has been used in a couple of previous studies for the partial enzymatic transesterification (i.e., selective ethanolysis) of sunflower oil for the production of Ecodiesel®-100, a mixture of two parts of fatty acid ethyl esters and one part of monoglyceride, that integrates the glycerol as a soluble derivative product in the diesel fuel [48].

**Table 1:** Biolipasa-R specifications, provided by the manufacturer Biocon S.L [47]

Parameter	Value
Lipolytic activity (FIP)	10,000
Temperature range (°C)	20–45
pH range	4.0–8.0

Luna et al. [49, 50] conducted an evaluation of the enzymatic ethanolysis reaction using Biolipasa-R, both in its commercially available free form and after immobilization on sepiolite through covalent bonding. In their studies, they optimized ethanolysis reaction parameters using Response Surface Methodology (RSM) and determined the optimal conditions, which comprised reaction temperature of 20°C, a 6:1 ethanol/oil molar ratio, pH 12.0, 0.15% water content (by weight of oil), and the lipase amount (by weight relative to the oil) in the range of 0.05–0.1%. For the immobilized enzyme, modifications were made to the temperature (30°C), pH (11.0), and lipase amount used (15% by weight of oil). Under these conditions, they achieved high biofuel yields, reaching up to 80% in a short time (1 h), and achieved excellent reusability of the immobilized enzyme, retaining almost 95% of its activity after five uses. Our work focused on the utilization of low cost Biolipasa-R and the assessment of its activity for the enzymatic transesterification of an alternative substrate (i.e., acid oil) employing methanol as the typical alkyl alcohol in biodiesel production.

In this study, response surface methodology (RSM) was applied aiming at the optimization of the enzymatic transesterification process of low-quality acid oil employing methanol and the Biolipasa-R. For this purpose, several factors influencing the transesterification process were examined, including enzyme concentration, water content, methanol to oil (molar) ratio, pH, and reaction temperature. The potential enhancement of transesterification efficiency by combining Biolipasa-R, a 1,3-regioselective lipase, with lipases exhibiting different regioselectivities under optimal conditions was also studied. Furthermore, *in situ* immobilization of Biolipasa-R was achieved through physical adsorption on Lifetech™ ECR8806M polymer beads (Purolite®), followed by a comparative assessment of transesterification efficiency between Biolipasa-R in its free and immobilized forms. In an effort to maximize FAME production during transesterification using the immobilized Biolipasa-R, factors such as temperature and the amount of water were examined. Finally, the reusability of the immobilized ROL was evaluated by employing the immobilized lipase at consecutive transesterification cycles.

## Materials and Methods

### Materials

Biolipasa-R (free enzyme, *Rhizopus oryzae* lipase, 1, 3-regioselective lipase) and Biolipasa-IN (immobilized *Candida antarctica* lipase B, non-regioselective lipase) were purchased from BIOCON (Barcelona, Spain). Lipozyme CALB L (free enzyme, *Candida antarctica* lipase B, non-regioselective lipase) and Lipozyme RM (immobilized *Rhizomucor miehei* lipase, 1,3-regioselective lipase) were purchased from STREM (Bischheim, France). The enzymes were stored refrigerated (4 °C), and their activity was periodically monitored, concerning the stability over time through a transesterification reaction at standard conditions; the observed activity change was found to be less than 6%.

The acid oil sample, a mixture of several batches of the byproduct from the chemical refining of edible oils, was kindly provided by Fytoenergeia S.A. (Serres, Greece), a biodiesel-producing company. The free fatty acid content of the acid oil sample was 18.05 mg KOH/g_oil_, determined according to EN14104, and the kinematic viscosity was found to be 47.013 cSt, determined by ASTM D7042 standard method, whereas the fatty acid profile, analyzed by GC-FID, is presented in Table S1.

Supelco 37 Component FAME Mix was of chromatographic grade and was purchased from Merck (Darmstadt, Germany). Methyl tricosanoate used as an internal standard was purchased from Sigma-Aldrich (Darmstadt, Germany). All other reagents and solvents used in this study were of analytical grade and were obtained from Merck (Darmstadt, Germany).

### Analysis of Fatty Acid Methyl Esters by GC-FID

The FAME content in the reaction mixture was determined following the EN14103 standard, using a gas chromatograph GCMS-QP2010 Ultra Gas chromatograph (Shimadzu Europe, GmbH), connected with an autosampler (AOC-20i Auto Injector, Shimadzu Europe, GmbH), with a SP-2340 capillary column (60 m × 0.25 mm, 0.20 μm film thickness) from Supelco (Bellefonte, PA, USA), and a flame ionization detector (FID). The temperatures of detector and injector were adjusted at 250°C. The column temperature was maintained at 100°C for 5 min, and then increased to 240°C at a rate of 4°C/min, and maintained at 240°C for 30 min. Helium was used as the carrier gas and methyl tricosanoate (C23) as an internal standard [51, 52].

The fatty acid methyl ester % content (FAME %) was calculated according to Eq. (1).

1 $$\mathrm{FAME}\ \left(\%\right)=\frac{SA-{A}_{IS}}{A_{IS}}\times \frac{C_{IS}\times {V}_{IS}}{m_{sample}}\times 100$$

where SA is the total peak area of FAME (C14:0 to C22:0); A_IS_ is the peak area of the internal standard; C_IS_ is the concentration of the internal standard (1 mg/mL); V_IS_ is the volume of the internal standard (0.3 mL), and m_sample_ is the sample mass (25 mg).

### Enzymatic Biodiesel Production and Optimization

The transesterification reactions were carried out in batch mode in 50 mL flasks, under heating and magnetic stirring. In the first series of experiments, Biolipasa-R was used. The transesterification reaction was performed by mixing a certain amount of acid oil, lipase, methanol, and water, and stirring at 180 rpm for 72 h. Methanol was added to the mixture in three steps at 0, 24, and 48 h. At the end of each batch, the resulting biodiesel (upper layer) was separated by centrifugation (4000 rpm, 10 min) and used for GC-FID analysis to determine the FAME content.

The study focused on individual and interactive effects of the following selected variables: (i) lipase concentration (20–60%, by weight of oil), (ii) water content (10–50 %, by weight of oil), (iii) methanol to oil (molar) ratio (3:1–6:1), and (iv) the pH value (5.0–7.0) on the FAME content (as response), using RSM experimental design at Design Expert® 13 Software (Stat-Ease Inc., Minneapolis, MN, USA). RSM provides a mathematical model that identifies the optimal combination of the chosen factors to enhance the selected response. This model elucidates the relationship between factors and the interaction between the combination of factors and response within the entire experimental domain [53]. A three-level, four-factor, face-centered central composite (FCC) design was employed in this study, totally requiring 52 experiments. The CCD design is valuable when examining systems with numerous factors and interactions. It consists of factorial, central, and axial points and has the capability to predict high-quality linear and quadratic models. In addition, it provides a well-balanced approach between the number of experiments conducted and the precision of the results obtained [53]. The range of each parameter, which was selected according to existing literature [11, 51] and to the results of preliminary experiments, is presented in Table 2.

**Table 2:** Level of values of the independent variables of the statistical design experiments

Level of values	Factor X_1_ Biolipasa-R (ROL) concentration (% w_ROL_/w_oil_)	Factor X_2_ Water concentration (% w_Water_/w_oil_)	Factor X_3_ MeOH:Oil (mol/mol)	Factor X_4_ pH
-1	20	10	3.0	5.0
0	40	30	4.5	6.0
1	60	50	6.0	7.0

Furthermore, in order to investigate the effect of temperature on transesterification process, experiments of the reaction were conducted at 26, 30, 35, 40, and 45°C, at the optimum transesterification conditions, as specified by the RSM experimental design. The time course (reaction kinetics) of Biolipasa-R catalyzed transesterification was also studied aiming to reduce the overall reaction time. The experiment was conducted in 150 mL flasks under optimal conditions. Samples (approximately 1 mL each) were collected at specific time points, i.e., every half-hour at the beginning of the reaction and subsequently every almost 2 h. These samples were analyzed using GC-FID to determine the FAME content.

All experiments were carried out in triplicate, and analysis of variance (ANOVA) was used to evaluate the statistical significance of the factors (statistical significance was considered at *p* value < 0.05).

### Synergistic Effect of Lipases on the Biodiesel Yield

The synergistic effect of two different lipases, whether in their free form, immobilized, or in combination thereof, was studied, aiming to maximize the production of FAME. A combination of lipases with different regioselectivity and the effect of varying mixing ratios between the regioselective and non-regioselective lipases has been evaluated under the optimum transesterification conditions, as specified by the RSM experimental design. The different combinations of lipases tested are summarized in Table 3.

**Table 3:** Transesterification experiments of acid oil using different lipases and combination thereof, in several mixing ratios by weight, under optimal conditions

Experiment	Lipases	Mixing ratios of lipases
1	Biolipasa-R	n.a.
2	Lipozyme CALB L	n.a.
3	Biolipasa IN	n.a.
4	Lipozyme RM	n.a.
5	Biolipasa-R : Lipozyme CALB L	1:1
6	Biolipasa-R : Lipozyme CALB L	2:1
7	Biolipasa-R : Lipozyme CALB L	3:1
8	Biolipasa-R : Lipozyme CALB L	4:1
9	Biolipasa-R : Lipozyme CALB L	5:1
10	Biolipasa-R : Biolipasa IN	3:1
11	Biolipasa-R : Biolipasa IN	4:1
12	Biolipasa-R : Biolipasa IN	5:1
13	Lipozyme RM : Biolipasa IN	3:1
14	Lipozyme RM : Biolipasa IN	4:1
15	Lipozyme RM : Biolipasa IN	5:1

### Immobilization of Biolipasa-R on Octadecyl Methacrylate

For the immobilization of Biolipasa-R via physical adsorption onto octadecyl methacrylate (Lifetech™ ECR8806M), 200 g Biolipasa-R (200 mg of the enzyme) was dissolved in 600 mL of 50 mM potassium phosphate buffer, pH 7.5 (KPi). The enzyme solution was then introduced into a 1 L SpinChem® rotating bed reactor containing 3 g of wet beads. A sample of the enzyme supernatant was extracted to measure the initial enzyme concentration using the Bradford method and the initial specific activity of the free enzyme through a *p*-nitrophenyl butyrate (pNPB) hydrolysis reaction. Following the initial sampling, the enzyme and carrier mixtures underwent gentle stirring at 150 rpm at room temperature for 24 h. To monitor the progress of immobilization, additional samples were collected at intervals of 1, 3, 5, and 24 h. Upon completion of the immobilization process, the enzyme solution was separated from the carrier and subjected to 3 washes with KPi buffer. Subsequently, the carrier was dried using a SpeedVac concentrator and stored in a vacuum desiccator at 8°C for future use.

Free enzyme activity was evaluated with pNPB as substrate. Briefly, pNPB stock solution (10 mm) was prepared in dimethyl sulfoxide (DMSO). The enzyme was properly diluted in pre-warmed (30°C) potassium phosphate buffer (50 mm, pH 7.5). pNPB solution (20 μL) was added in a 96-well plate and mixed with 160 μL of preheated potassium phosphate (KPi) buffer (50 mM, pH 7.5) until homogeneous. The reaction was initiated with the addition of 20 μL of enzyme solution. The reaction rate was monitored spectrophotometrically at 405 nm for 5 min at 30°C, by following the *p*-nitrophenol (pNP) release. All measurements were performed (at least in triplicates) on a UV/VIS Multiskan Sky Microplate photometer. One Unit was defined as the amount of lipase required to release 1 μmol of pNP per minute under the given conditions.

### Efficiency and Reusability of Immobilized Biolipasa-R

The transesterification reactions using immobilized Biolipasa-R were carried out in 50 mL flasks on a heating magnetic stirrer under optimal experimental conditions derived from the optimization experiments performed using the enzyme in its free form. Specifically, the transesterification mixture was comprised of a certain amount of acid oil, immobilized enzyme concentration corresponding to the specific activity of free lipase, methanol (3:1 molar ratio of methanol to oil), water concentration of 32% per weight of oil and pΗ of 7.0. Two different temperatures were tested at this point, initially at 30°C, and then at 37°C. Methanol was also added to the mixture in three steps, at 0, 6 and 24 h. The transesterification mixture was stirred at 180 rpm, while the reaction duration was 48 h. The addition of a lower amount of water (16% and 2% per weight of oil) in the transesterification reaction (in the case of immobilized Biolipasa-R), was further investigated, at the temperature that yielded the highest FAME content.

The immobilized Biolipasa-R was also evaluated concerning its reusability for biodiesel production. Immobilized lipase beads were separated from the reaction mixture by centrifugation, then washed twice with deionized water after each transesterification cycle, and directly reused into a new transesterification experiment. No new lipase was added during the reusability tests that lasted for three transesterification cycles.

## Results and Discussion

### Optimized Transesterification Parameters by RSM

**Fig. 1:**
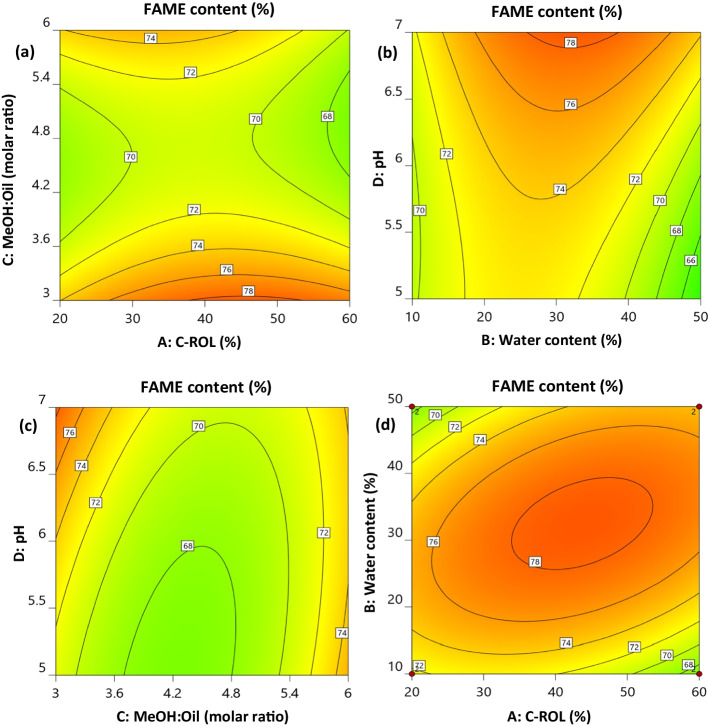
Contour plots of FAME content (%) as a function of lipase concentration and methanol:oil molar ratio (**a**), water content and pH value (**b**), methanol:oil molar ratio and pH value (**c**), and lipase concentration and water content (**d**)

For the experimental design and optimization of the enzymatic transesterification of acid oil, using Biolipasa-R, the Design Expert® v13 software was used. Fifty-two (52) experiments in total were performed, which are summarized in Table S2. To evaluate the interactive effects of the four independent parameters studied, contour surface plots were designed and shown in Fig. 1. Figure 1a represents the combined effects of the lipase concentration on the reaction mixture and the methanol to oil molar ratio, Fig. 1b shows the combined effects of water content and pH value, Fig. 1c depicts the combined effect of methanol to oil molar ratio and pH value, and Fig. 1d shows the combined effects of the amount of enzyme and the amount of water added to the reaction.

From these plots, it is possible to observe the increase in the FAME content by increasing the concentration of enzyme added to the reaction mixture up to the value of 42% w/w, enzyme per weight of acid oil. The FAME content increased along with the increase in the amount of lipase in the reaction. The more lipase available to the process, the more substrate molecules could be incorporated into the active center of the lipase. Further increase of the lipase concentration leads to a progressive decrease of biodiesel yield. At this point, it is important to clarify that the amount of Biolipasa-R added (42% w/w) refers to the quantity of lyophilized powder. Specifically, each gram of lyophilized powder contains approx. 0.03 grams of proteins (i.e., enzymes).

As can be seen from Fig. 1d, similar trends are observed regarding the water addition to the system. Increasing the water amount up to a concentration of 32% w/w per weight of acid oil and the concentration of lipase up to 42% w/w enzyme per weight of acid oil resulted in an increase in FAME content. However, further increases in both values led to a decrease in FAME content. Water presence influences the activity and stability of the free enzyme. However, an excessive amount of water could increase the flexibility of the lipase, also resulting in unintended side reactions, such as hydrolysis, particularly during the transesterification process [54]. Based on these results, it is evident that free Biolipasa-R exhibits reduced activity at lower water content during transesterification reaction. These findings are in accord with those presented by Wang et al. [55], who used the same type of lipase. They observed that FAME production using free *Rhizopus oryzae* lipase increases with an increasing amount of water, up to the value of 80% w/w [55]. Furthermore, in another study involving the transesterification of waste cooking oil samples using free *Rhizopus oryzae* lipase, water content of 50% w/w was recommended [56]. This recommendation was made due to the higher viscosity of the waste cooking oil sample, similar to the acid oil sample used in this study, aiming to ensure a greater oil-water interface area at which lipase displays activity. Furthermore, the low purity of Biolipasa-R may be another reason for the higher requirement of water addition in the reaction.

Concerning the pH of the reaction, as illustrated in Figs. 1b and 1c, FAME content increased sharply by increasing the pH value. At higher pH values (around 7.0) it is possible to obtain higher FAME yields at a methanol:oil molar ratio 3:1, a trend that is also reported on the literature [15, 55]. On the other hand, the increase of methanol in the reaction mixture, above the 3:1 ratio, inhibits the performance, as the excess of methanol possibly leads to lipase denaturation and deactivation [57]. Therefore, a step-wise methanol addition strategy is commonly applied [55, 58]. It is noteworthy that in our study, an excess of methanol was avoided since methanol was added to the reaction stoichiometrically (i.e., methanol reacts with triglycerides in 3:1 molar ratio). This approach is also beneficial in terms of the economic feasibility of the process by reducing the need for subsequent methanol recovery. Luna et al. [49] utilized the same lipase, Biolipasa-R, to optimize the enzymatic ethanolysis of sunflower oil. In their study, ethanol was used, and the optimization of reaction parameters through Response Surface Methodology (RSM) revealed that the optimum transesterification pH was considerably higher (pH=12.0) compared to our findings (pH=7.0). Additionally, they achieved the maximum ester yield (up to 80%) using a higher molar ratio of ethanol to oil (6:1). However, in their experiments, Biolipasa-R exhibited better performance with a significantly lower water concentration, specifically 0.15% per weight of oil.

Table 4 describes the optimum conditions for the maximum produced FAME during the biocatalytic transesterification of acid oil. By carrying out 52 experiments on various values of the selected parameters, the maximum yield of 75.5% was predicted under the given set (Table 4) of reaction conditions at 72 h reaction time and 30°C temperature. The low purity of Biolipasa-R consequently requires the use of a relatively high amount of lyophilized powder, which may be restrictive in large-scale applications of this lipase. However, considering that Biolipasa-R is 1, 3-regioselective, FAME production at these conditions reached its maximum. In their study, Li et al. [21] addressed the kinetic study of *Rhizopus oryzae* lipase methanolysis of triglycerides and the potential acyl migration, leading to yields higher than 66% FAME. They concluded that the examined intracellular lipase of *R. oryzae* did not exhibit strict selectivity for the 1(3)-position acyl but rather a preference for the 1(3)-position over the 2-position, which is quite promising. In our case, the remaining percentage (approx. 25%), except FAME, may be considered as triglycerides, partially reacted di- and mono-glycerides, as well as traces of glycerol, methanol, and water. These components can be separated in an industrial process through physicochemical steps such as washing, centrifugation, and/or FAME distillation.

**Table 4:** Optimum transesterification conditions for biodiesel production of acid oil using Biolipasa-R

Parameter	Value
Lipase concentration (% w/w, per weight of oil)	42
Water content (% w/w, per weight of oil)	32
Methanol to oil ratio (molar ratio)	3:1
pH	7.0

After fitting the results presented in Table S2, a quadratic model was depicted to be the most suitable one with *p* value < 0.05. The model equation in terms of coded values is represented by Equation (2):

2 $$\mathrm{FAME}\ \mathrm{Content}\ \left(\%\right)=67.46+0.4111\cdot {\mathrm{X}}_1+3.32\cdot {\mathrm{X}}_2-1.12\cdot {\mathrm{X}}_3+1.21\cdot {\mathrm{X}}_4+2.96\cdot {\mathrm{X}}_1\cdot {\mathrm{X}}_2-1.77\cdot {\mathrm{X}}_1\cdot {\mathrm{X}}_3-1.53\cdot {\mathrm{X}}_1\cdot {\mathrm{X}}_4+4.38\cdot {\mathrm{X}}_2\cdot {\mathrm{X}}_3+1.99\cdot {\mathrm{X}}_2\cdot {\mathrm{X}}_4-2.00\cdot {\mathrm{X}}_3\cdot {\mathrm{X}}_4-2.72\cdot {\mathrm{X}}_{1^2}-5.58\cdot {\mathrm{X}}_{2^2}+5.79\cdot {\mathrm{X}}_{3^2}+0.8861\cdot {\mathrm{X}}_{4^2}$$

According to the above equation, the influence of transesterification conditions, by comparing each factor’s coefficients, increased in the order of enzyme concentration (X_1_), methanol to oil molar ratio (X_3_), pH (X_4_), and water content (X_2_). Moreover, concerning the interactions, the water content with the methanol to oil molar ratio (X_2_·X_3_) interaction had the highest influence, followed by the interaction of enzyme concentration with the water content (X_1_·X_2_), and by the interactions of methanol to oil molar ratio with pH (X_3_·X_4_) and of water content with the pH (X_2_·X_4_). In addition, an F-value of 7.97 and a p-value less than 0.0001, according to ANOVA analysis performed, indicating that the model is significant. Additionally, based on the Lack of Fit F-value, which is 1.82, it is evident that Lack of Fit is not significant relative to pure error. Apart from that, a coefficient of variation (CV) of 6.50% underscores the precision and reliability of the experiments conducted. Generally, when the coefficient of variation is below 10%, the model is considered to be reasonably reproducible.

To assess the accuracy of the predictions, a triplicate experiment was performed under the optimum conditions. The obtained average yield, 71.3 ± 0.6%, closely matched the predicted value of 75.5 %. The percent convergence at the optimum conditions was approx. 94.4%, which is considered acceptable. Therefore, the predictions are confirmed as accurate and reliable for estimating the response variable.

### Effect of Reaction Temperature

**Fig. 2:**
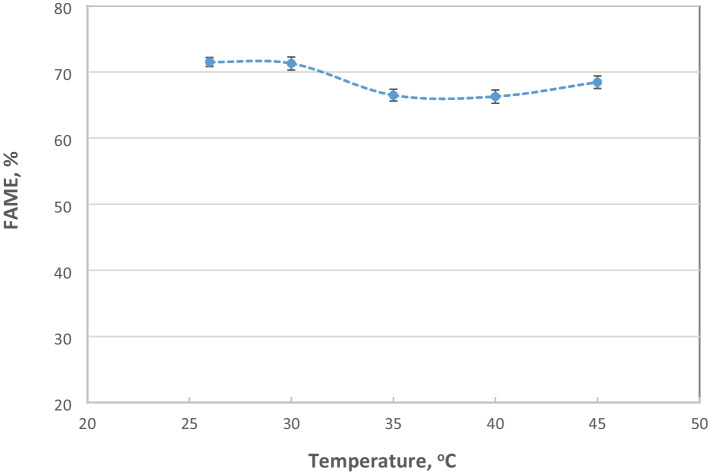
Effect of reaction temperature on FAME production (%). Reaction conditions: enzyme concentration 42% w/w, per weight of oil, 32% w/w water content (per weight of oil), methanol:oil 3:1 (mol/mol), pH 7.0 and agitation at 180 rpm, for 72 h

The impact of temperature on acid oil transesterification using Biolipasa-R was investigated within the enzyme’s specified functional temperature range according to the manufacturer’s instructions (Table 1). The results, as illustrated in Fig. 2, show that the optimum temperature for this lipase was 30°C; however, the effect of temperature on FAME content is rather insignificant. Slightly lower FAME content was obtained at a reaction temperature of 26°C, followed by a gradual decrease in FAME content until 40°C. Although there was a slight increase in FAME produced at 45°C, the content remained lower than the one achieved at 30°C, which was consequently selected as the optimal temperature. These findings are consistent with those reported by Wang et al. [55], who conducted a similar study on the effect of temperature on ROL both in free and immobilized forms. Slightly higher ester yield at 20°C compared to that at 30°C were reported by Luna et al. [50] during the ethanolysis reaction of sunflower oil using Biolipasa-R.

### Kinetics of FAME Production at the Optimum Reaction Conditions

**Fig. 3:**
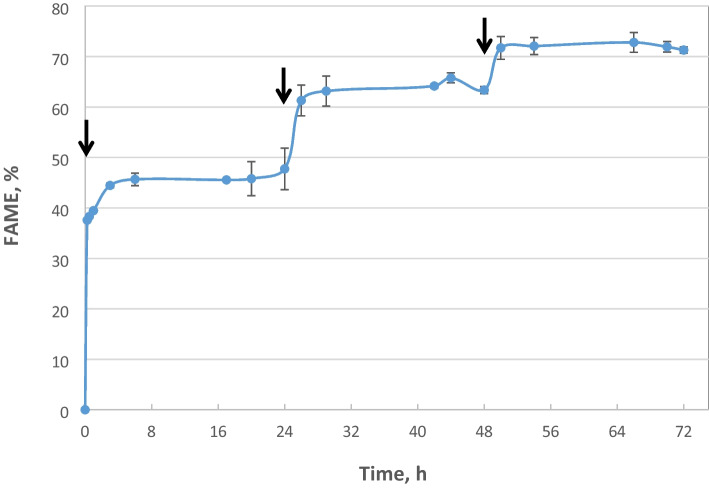
FAME production (%) versus reaction time. Reaction conditions: enzyme concentration 42% w/w by weight of oil, 32% w/w water content by weight of oil, methanol:oil 3:1 (mol/mol), pH 7.0, and agitation at 180 rpm for 72 h. Methanol addition time points are indicated by black arrows

Figure 3 shows the kinetics of FAME production obtained at 30°C under optimum conditions. The experiment was carried out by three sequential additions of methanol at 0, 24, and 48 h of reaction. Based on the results obtained, the transesterification reaction kinetics reached a plateau 6 h after the first and second methanol addition to the reaction mixture and after 2 h after the third methanol addition. Based on these results, the reaction duration was shortened according to the following protocol: the first 1/3 of the total methanol amount was added to the reaction mixture at the beginning, followed by a second addition (1/3) after 6 h, and lastly, a third addition after 12 h, while the reaction was terminated after approx. 15 h, resulting in the same high FAME yield. Furthermore, the use of three molar equivalents of methanol in these experiments led to the highest reaction yield, even though it corresponds to the minimum stoichiometric amount of methanol needed for complete FAME conversion.

### Synergistic Effect of Commercial Lipases

**Fig. 4:**
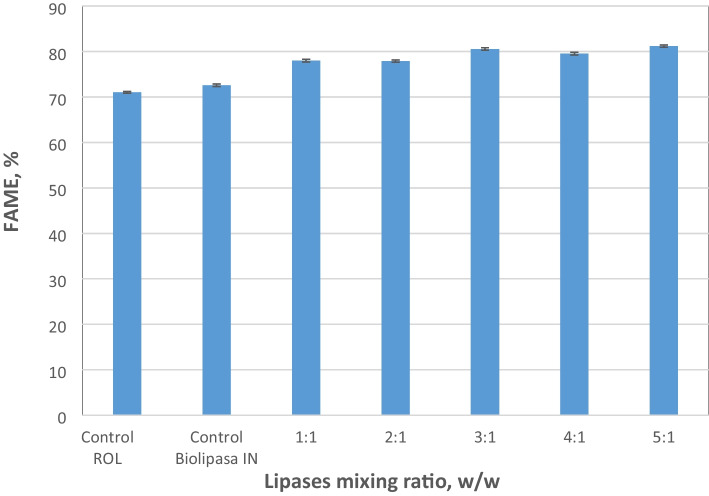
Effect of Biolipasa-R (ROL) and Biolipasa-IN mixing ratio, from 5:1 to 1:1 weight ratios, on acid oil transesterification under optimum conditions (total enzyme concentration 42% w/w per weight of oil, 32% w/w water content per weight of oil, methanol:oil 3:1 (mol/mol), pH 7.0, temperature 30°C and agitation at 180 rpm, for 48 h)

In the present experiments, two different commercial lipases, Lipozyme CALB L (free lipase) and Biolipasa-IN (immobilized lipase), were examined as non-regioselective lipases in combination with Biolipasa-R. The effect of different mixing ratios between Biolipasa-R and each one of the non-regioselective lipases has also been evaluated, while the total amount of enzymes was maintained at a concentration of 42% per weight of oil. Figure 4 shows that the highest FAME content was achieved at a mixing ratio of 5:1 Biolipasa-R (ROL) and Biolipasa-IN. Specifically, at this lipase ratio, the increase in FAME produced was around 14% and reached a content of 81.2±0.2%. This result is in accordance with the finding of Su et al. [59], who also highlighted the synergistic effect of ROL when combined with Novozyme 435. Novozyme 435, derived from *Candida antarctica* B, is a non-regioselective lipase similar to Biolipasa-IN, with their main distinction being the immobilization carrier used. In their study, they investigated soybean oil transesterification using a 3:1 mixing ratio of ROL and Novozyme 435, resulting in an 88.2% FAME production yield [59]. In contrast to these findings, no synergy appears when coupling ROL and Lipozyme CALB L. The latter lipase is the free lipase B of *Candida antarctica* and resulted in a FAME content of 64.6±0.2%, which was lower than the conversion achieved with Biolipasa-R (71.3±0.6%) alone. CALB L did not present any significant result (48.3±0.5%) indicating that, unlike its immobilized form, the free form of the lipase was not suitable for the transesterification of this kind of oils. Similar findings have been reported in the literature regarding the transesterification of refined soybean oil and acid oil using CALB L, which yielded a maximum of 2% and 32%, respectively. These results were obtained at a temperature of 30°C after 4 h of transesterification [60]. Additionally, Duleba et al. [61] noted that CALB L exhibits its highest activity in non-polar organic solvents, such as hexane, while demonstrating the lowest activity in polar solvents, such as methanol.

Apart from the use of Biolipasa-R, Lipozyme RM (immobilized, 1,3-regioselective lipase) was also combined with Biolipasa-IN (which yielded the best results in the aforementioned experiments) to assess the impact of different mixing ratios between Lipozyme RM and Biolipasa-IN. At a mixing ratio of 3:1 (Lipozyme RM and Biolipasa-IN), the performance was even better than that of Biolipasa-R when combined with Biolipasa-IN. Compared with the single lipases, the combined strategy achieved over 20% enhancement in biodiesel yield (Table 5), which is in accord with results from other studies [1, 12, 59] that achieved the highest biodiesel yield (93.1±0.2%) by combining immobilized *C. rugosa* and *R. miehei* in a 1:1 mixing ratio, while the use of single *C. rugosa* led to a yield of 83%. Ecodiesel®, a biofuel similar to biodiesel that keeps glycerol as monoglycerides along with two fatty acid ethyl esters (FAEE) molecules, was obtained through a selective ethanolysis process of sunflower oil, employing Lipozyme RM [62] and Novozym 435 [63], which is an enzyme similar to Biolipasa-IN. Lipozyme RM and Novozym 435 resulted in FAME content of 89% and 51%, respectively [62, 63]. In our study, transesterification using these lipases was evaluated for biodiesel production under milder conditions, such as lower pH (7.0) and temperature (30°C), and by employing a stoichiometric ratio of methanol (3:1 methanol:oil), aiming to maintain an economically viable process.

**Table 5:** Maximum FAME yield of acid oil transesterification using single lipases and combinations thereof at varying mixing ratios, using a total enzyme concentration of 42% w/w per weight of oil

Lipases / mixing ratios of lipases	FAME yield (%)
Biolipasa-R (ROL)	71.3 ± 0.6
Lipozyme CALB L	48.3 ± 0.5
Biolipasa-IN	72.6 ± 0.3
Lipozyme RM	76.0 ± 0.4
Biolipasa-R (ROL) : Lipozyme CALB L - 2:1	64.6 ± 0.2
Biolipasa-R (ROL) : Biolipasa-IN - 5:1	81.2 ± 0.2
Lipozyme RM : Biolipasa-IN - 3:1	90.1 ± 0.4

### Immobilization of Biolipasa-R on Octadecyl Methacrylate

**Fig. 5:**
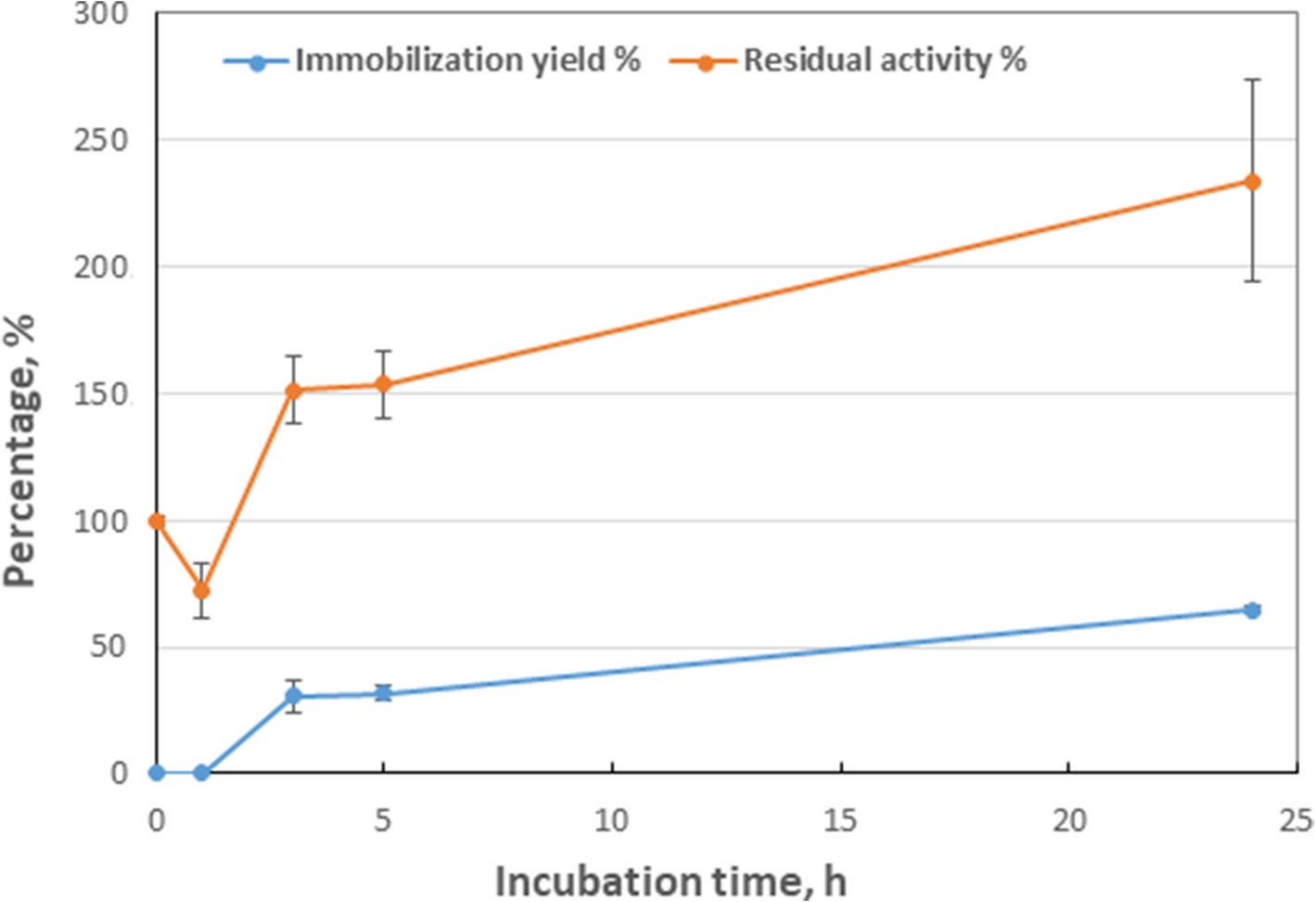
Immobilization yield (%) and residual activity (%) of Biolipasa-R on octadecyl methacrylate, through physical adsorption

The immobilization experiment involving Biolipasa-R on octadecyl methacrylate yielded promising outcomes, as evidenced by Fig. 5. Following a 24 h incubation of the enzyme with the carrier, an apparent immobilization rate of approximately 65% was observed. Kinetic measurements conducted on samples extracted at distinct time points (0, 1, 3, 5, and 24 h) determined the percentage of remaining enzyme activity, as depicted in Fig. 5. Analysis of the residual activity of Biolipasa-R throughout the immobilization procedure revealed a progressive increase at 234%. This amount surpasses the initial activity level (100%) more than twofold. This encouraging result underscores the efficacy of the immobilization process under the prevailing conditions.

Additionally, experiments were performed to measure the specific activity of Biolipasa-R following immobilization on octadecyl methacrylate, employing pNPB hydrolysis reactions. The outcome of these investigations revealed a specific activity of 0.49 ± 0.01 U/mg of immobilized enzyme. The decreased specific activity observed in the immobilized Biolipasa-R can be attributed to several factors, including diffusion and mass transfer limitations [64]. The immobilization process may be challenging as the enzyme’s active site becomes obstructed by the carrier, resulting in low substrate interaction. Additionally, the immobilized enzymes may encounter mass transfer limitations, particularly when substrates or products transport to or from the active sites within the immobilized matrix, thus contributing to the reduction of specific activity.

### Efficiency and Reusability of Immobilized Biolipasa-R

**Fig. 6:**
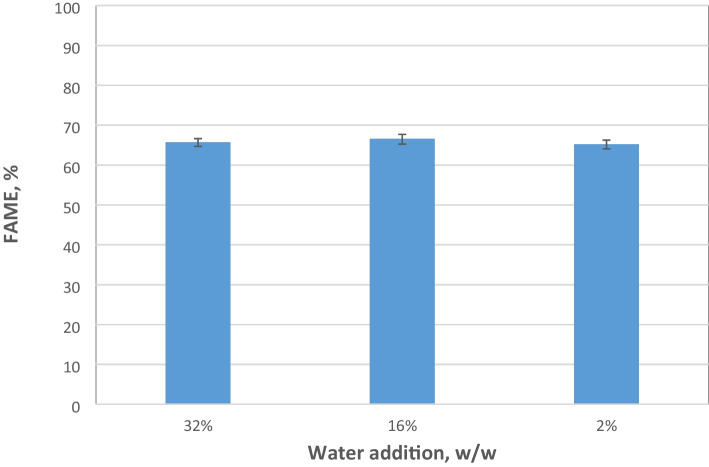
Effect of added water on FAME yield (%) using immobilized Biolipasa-R. Reaction conditions: acid oil, methanol to oil molar ratio 3:1, enzyme dosage 5.12 U, pH 7, T = 37°C and 180 rpm for 48 h

Experiments were also performed to assess the transesterification efficiency of immobilized Biolipasa-R onto octadecyl methacrylate at two different temperatures (30 and 37°C) and three water contents (2%, 16%, and 32% per weight of oil). It was observed that the immobilized lipase exhibited better performance at 37°C (66.5±1.2%) while maintaining all other parameters at the optimum values obtained using the free Biolipasa-R. These findings align with the results of another study, which concluded that immobilized ROL in various carrier resin performed better at 37°C [55], in contrast to the free enzyme that the best yield was achieved at 30°C. Results presented in Fig. 1 show that the free Biolipasa-R activity was poor at low water content, while results in Fig. 6 indicate that immobilized Biolipasa-R performed satisfactorily even with rather low water addition in the transesterification mixture.

**Fig. 7:**
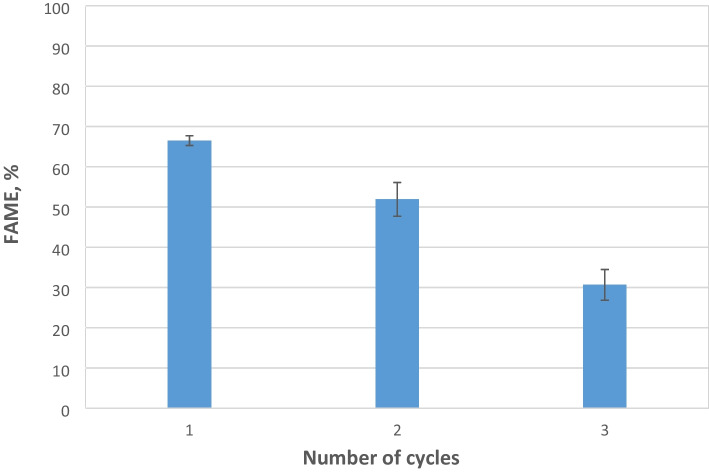
Reusability of immobilized Biolipasa-R for repeated batch transesterification reaction. Reaction conditions: acid oil, methanol to oil molar ratio 3:1 enzyme dosage 5.12 U, 16% w/w water by weight of oil, pH 7, T = 37°C and 180 rpm for 48 h

The reusability of enzymes is a critical parameter in the transesterification process for biodiesel production. The high cost of enzymes is closely linked to the economic feasibility of the process, which can be significantly enhanced by the ability to reuse the lipase for multiple reactions [65]. The immobilized Biolipasa-R was washed with de-ionized water after each reaction and used in the next reaction cycle using the new substrate. The results presented in Fig. 7 indicate that the FAME yield after the first reaction, under optimized parameters, was 66.5±1.2%, while after the second cycle of reaction, the enzyme was still effective and retained almost 78% of its initial activity. However, at the third use, the yield decreased, and immobilized Biolipasa-R had lost over 50% of its activity.

The decrease in transesterification yield was a consequence of the reduced activity of the immobilized lipase. This reduction can be partly attributed to the potential desorption of the lipase from the adsorbent. In our study, Biolipasa-R was immobilized onto an octadecyl methacrylate support using physical adsorption technology. A similar study conducted by Wang et al. [55] concluded that both physical and chemical adsorption methods were less effective compared to covalent binding technology, and they also noted that after four uses, immobilized ROL through physical adsorption onto a macroporous resin gradually lost its activity. Luna et al. [50] also suggested that covalently immobilization of Biolipasa-R onto sepiolite (using a p-hydroxybenzaldehyde linker) resulted in better stability, retaining almost 100% of its activity until the fifth use. However, when Biolipasa-R was immobilized by physical entrapment on demineralized sepiolite, a gradual decrease in activity starting from the first reuse was exhibited [50]. Additionally, this reduction in activity, also reported in similar studies [57, 65, 66], may be due to the deactivation or denaturation of the enzyme after many cycles of use or due to repeated exposure of the enzyme to methanol present in the reaction mixture.

Immobilization of Biolipasa-R on octadecyl methacrylate offers several advantages, along with some limitations and potential improvements to enhance the recyclability of the catalyst [67]. Regarding the advantages, first, we get a simplified downstream process. Immobilization simplifies downstream processing by eliminating the need for extensive purification steps, reducing the time required for catalyst recovery [20]. Potential improvements in the reusability of the catalyst have to do with the optimization of immobilization conditions and methods. Fine-tuning immobilization conditions such as enzyme concentration, immobilization time, and support surface area can optimize the loading efficiency and catalytic performance of the immobilized catalyst. Regarding the immobilization method, we could proceed with experiments about covalent immobilization that will prevent enzyme leaching and increase recyclability [68]. This strategy entails utilizing glutaraldehyde for covalent immobilization, fostering strong chemical bonds between the amino-functionalized carrier and the enzyme. Moreover, recent research has concentrated on immobilizing lipases onto commercially available or modified membranes with promising outcomes, demonstrating enhanced stability of the immobilized enzyme even after multiple transesterification cycles [20, 69].

## Conclusions

This work focuses on the enzymatic transesterification of low-quality acid oil for biodiesel production. A low-cost, commercially available lipase, Biolipasa-R was used for the optimization of transesterification conditions carried out through Response Surface Methodology (RSM). The highest biodiesel yield of 71.3% was achieved within 48 h under specific conditions: 42% lipase concentration (per weight of oil), 32% water content (per weight of oil), a methanol to oil molar ratio of 3:1, pH 7.0 and reaction temperature 30°C. To enhance the conversion yield, a combination of lipases with different regioselectivities was explored for their synergistic effect, and the mixture of Lipozyme RM and Biolipasa IN at a 3:1 ratio yielded the highest FAME percentage at 90.1%. Therefore, using lipase mixtures could be a promising alternative for biodiesel production. In addition, the immobilization of Biolipasa-R through physical adsorption was evaluated for transesterification efficiency, achieving a maximum yield of 66.5%. However, the reusability study indicated that the immobilized enzyme retained its satisfactory activity for only two consecutive cycles. Thus, further investigation on the immobilization carrier and an appropriate immobilization method are necessary for potential industrial applications in biodiesel production. Despite this limitation, immobilized low-cost Biolipasa-R offers advantages, such as easy recovery, making it a promising biocatalyst for biodiesel production in a relatively cost-effective manner.

## Supplementary Information

ESM 1 (DOCX 33 kb)

## Data Availability

All data generated during this study are included in this published article and its supplementary information files.
