# Supplementary material for: Optimization of Enzymatic Transesterification of Acid Oil for Biodiesel Production Using a Low-Cost Lipase: The Effect of Transesterification Conditions and the Synergy of Lipases with Different Regioselectivity
Source: Appl Biochem Biotechnol. 2024 May 2;196(11):8168–89. doi: 10.1007/s12010-024-04941-3 (PMC11645316; doi:10.1007/s12010-024-04941-3)
Supplement: Supplementary file 1 — (DOCX 33 kb) [file 12010_2024_4941_MOESM1_ESM.docx]

**Optimization of enzymatic transesterification of acid oils for biodiesel production using a low-cost lipase: the effect of transesterification conditions and the synergy of lipases with different regioselectivity**

Alexandra Moschona^1^, Androniki Spanou^2^, Ioannis V. Pavlidis^2^, Anastasios J. Karabelas^1^ and Sotiris I. Patsios^1,*^

^1^ Laboratory of Natural Resources and Renewable Energies, Chemical Process and Energy Resources Institute, Centre for Research and Technology - Hellas, Thermi, Thessaloniki, Greece

^2^ Department of Chemistry, University of Crete, Heraklion, Greece

^*^ Corresponding author: [patsios@certh.gr](mailto:patsios@certh.gr)

A. Moschona: ORCID 0009-0007-3465-0633

I.V. Pavlidis: ORCID 0000-0001-5811-368X

A.J. Karabelas: ORCID 0000-0003-1463-519X

S.I. Patsios: ORCID 0000-0002-7180-9291

**Table S1.** Fatty acid composition of acid oil sample

| Peak Number | Compound Name | Area% |
| --- | --- | --- |
| 1 | C14:0 Myristic acid | 0.08 |
| 2 | C15:0 Pentadecanoic acid | 0.02 |
| 3 | C16:0 Palmitic acid | 6.87 |
| 4 | C16:1 Palmitoleic acid | 0.15 |
| 5 | C17:0 Heptadecanoic acid | 0.04 |
| 6 | C17:1 cis-10-Heptadecenoic acid | 0.04 |
| 7 | C18:0 Stearic acid | 2.75 |
| 8 | C18:1n9c Oleic acid | 36.50 |
| 9 | C18:2n6c Linoleic acid | 49.41 |
| 10 | C20:0 Arachidic acid | 0.30 |
| 11 | C20:1n9 cis-11-Eicosenoic acid | 0.33 |
| 12 | C18:3n3 Linolenic acid | 1.58 |
| 13 | C21:0 Heneicosanoic acid | 0.01 |
| 14 | C20:2n6 cis-11,14-Eicosadienoic acid | 0.02 |
| 15 | C22:0 Behenic acid | 0.69 |
| 16 | C22:1n9 Erucic acid | 0.05 |
| 17 | C23:0 Tricosanoic acid | 0.03 |
| 18 | C20:5n3 cis-5,8,11,14,17-Eicosapentaenoic acid | 0.21 |
| 19 | C24:1n9 Nervonic acid | 0.01 |

**Table S2.** Face-Centered Composite (FCC) design matrix for transesterification experiments employing Biolipasa-R and the observed response (% FAME content)

| Exp. | Factor X_1_  Biolipasa-R (ROL) Concentration  (% w_ROL_/w_oil_) | Factor X_2_  Water Concentration  (% w_Water_/w_oil_) | Factor X_3_  MeOH:Oil (mol/mol) | Factor X_4_  pH | Response Y_1_  FAME content (%) |
| --- | --- | --- | --- | --- | --- |
| 1 | 40 | 30 | 4.5 | 7 | 68.84 |
| 2 | 20 | 10 | 3.0 | 7 | 71.32 |
| 3 | 20 | 50 | 6.0 | 7 | 75.54 |
| 4 | 40 | 30 | 3.0 | 6 | 76.76 |
| 5 | 40 | 30 | 4.5 | 7 | 71.36 |
| 6 | 20 | 50 | 3.0 | 5 | 59.51 |
| 7 | 20 | 50 | 6.0 | 5 | 67.28 |
| 8 | 20 | 10 | 6.0 | 5 | 66.11 |
| 9 | 60 | 50 | 3.0 | 7 | 71.86 |
| 10 | 60 | 50 | 3.0 | 5 | 68.44 |
| 11 | 60 | 10 | 6.0 | 7 | 43.03 |
| 12 | 20 | 10 | 6.0 | 7 | 49.80 |
| 13 | 60 | 50 | 3.0 | 5 | 71.07 |
| 14 | 60 | 10 | 6.0 | 7 | 45.42 |
| 15 | 40 | 30 | 4.5 | 6 | 68.37 |
| 16 | 40 | 50 | 4.5 | 6 | 63.97 |
| 17 | 60 | 10 | 3.0 | 5 | 69.06 |
| 18 | 60 | 10 | 6.0 | 5 | 58.94 |
| 19 | 40 | 30 | 6.0 | 6 | 73.20 |
| 20 | 60 | 50 | 6.0 | 7 | 75.24 |
| 21 | 60 | 30 | 4.5 | 6 | 65.62 |
| 22 | 40 | 10 | 4.5 | 6 | 61.21 |
| 23 | 20 | 50 | 6.0 | 7 | 71.81 |
| 24 | 20 | 50 | 6.0 | 5 | 66.16 |
| 25 | 60 | 10 | 3.0 | 7 | 72.42 |
| 26 | 20 | 50 | 3.0 | 5 | 55.21 |
| 27 | 60 | 50 | 6.0 | 5 | 68.39 |
| 28 | 40 | 30 | 6.0 | 6 | 73.68 |
| 29 | 20 | 10 | 6.0 | 7 | 66.98 |
| 30 | 20 | 50 | 3.0 | 7 | 66.18 |
| 31 | 40 | 30 | 4.5 | 6 | 68.93 |
| 32 | 40 | 10 | 4.5 | 6 | 60.95 |
| 33 | 20 | 50 | 3.0 | 7 | 66.17 |
| 34 | 40 | 30 | 4.5 | 6 | 66.16 |
| 35 | 60 | 10 | 3.0 | 7 | 65.49 |
| 36 | 60 | 50 | 3.0 | 7 | 71.07 |
| 37 | 20 | 30 | 4.5 | 6 | 62.49 |
| 38 | 20 | 10 | 6.0 | 5 | 63.85 |
| 39 | 60 | 30 | 4.5 | 6 | 65.81 |
| 40 | 20 | 30 | 4.5 | 6 | 65.84 |
| 41 | 40 | 50 | 4.5 | 6 | 62.21 |
| 42 | 60 | 10 | 3.0 | 5 | 64.13 |
| 43 | 60 | 50 | 6.0 | 5 | 73.24 |
| 44 | 40 | 30 | 4.5 | 6 | 63.87 |
| 45 | 20 | 10 | 3.0 | 5 | 61.57 |
| 46 | 40 | 30 | 4.5 | 5 | 59.59 |
| 47 | 20 | 10 | 3.0 | 7 | 77.25 |
| 48 | 40 | 30 | 4.5 | 5 | 74.41 |
| 49 | 40 | 30 | 3.0 | 6 | 70.16 |
| 50 | 60 | 10 | 6.0 | 5 | 59.00 |
| 51 | 20 | 10 | 3.0 | 5 | 62.20 |
| 52 | 60 | 50 | 6.0 | 7 | 81.84 |
